# Supplementary material for: Y chromosome toxicity does not contribute to sex-specific differences in longevity
Source: Nat Ecol Evol. 2023 Jun 12;7(8):1245–56. doi: 10.1038/s41559-023-02089-7 (PMC10406604; doi:10.1038/s41559-023-02089-7)
Supplement: Supplementary file 1 — Full genotypes list. [file 41559_2023_2089_MOESM1_ESM.pdf]

nature ecology & evolution

Article

https://doi.org/10.1038/s41559-023-02089-7

Y chromosome toxicity does not contribute to sex-specific differences in longevity

In the format provided by the authors and unedited

Supplementary information. Full genotypes

Figure 1.

Figure 1C.

w;nubbin-Gal4 (BL#67086)/+;UAS-Cas9.P2 (BL#67086)/UAS-gRNA x223*

Figure 1D.

w/Dp(1;Y)B[S](BL#81622);UAS-Cas9.P2 (BL#58985)/+;eyeless-Gal4/+*
w/Dp(1;Y)B[S](BL#81622);UAS-Cas9.P2 (BL#58985)/+;eyeless-Gal4/UAS-gRNA x14*
w/Dp(1;Y)B[S](BL#81622);UAS-Cas9.P2 (BL#58985)/+;eyeless-Gal4/UAS-gRNA x27*
w/Dp(1;Y)B[S](BL#81622);UAS-Cas9.P2 (BL#58985)/+;eyeless-Gal4/UAS-gRNA x66*
w/Dp(1;Y)B[S](BL#81622);UAS-Cas9.P2 (BL#58985)/+;eyeless-Gal4/UAS-gRNA x223*

Figure 1E.

Males were obtained from the following crosses:
Canton-S females x C(1;Y)1, y^1, Bar^1,Y* males (Y* being: Y21, Y26, Y53, Y69 or Y72)
Note that C(1;Y)1, y^1, Bar^1 and each Y* chromosome were backcrossed eight generations into the laboratory's own Canton-S genetic stock.
X/Y21
X/Y26
X/Y53
X/Y69
X/Y72

Figure 1F.

Males were obtained from the following crosses:
Canton-S females x C(1;Y)1, y^1, Bar^1,Y* males (Y* being: Y100, Y53, or Y72)
Note that C(1;Y)1, y^1, Bar^1 and each Y* chromosome were backcrossed eight generations into the laboratory's own Canton-S genetic stock. Y100 correspond to the Y chromosome of the following stock: BL#78567 and carries a Y-linked RFP transgene. All the Y deletions of our library (Y21, Y26, Y53, Y69 or Y72) are derived from Y100.
X/Y100
X/Y53
X/Y72

Figure 1G.

Males were obtained from the following crosses:
Canton-S females x C(1;Y)1, y^1, Bar^1,Y* males (Y* being: Y100, Y21, Y26, Y53, Y69 or Y72)
Note that C(1;Y)1, y^1, Bar^1 and each Y* chromosome were backcrossed eight generations into the laboratory's own Canton-S genetic stock. Y100 correspond to the Y chromosome of the following stock: BL#78567 and carries a Y-linked RFP transgene. All the Y deletions of our library (Y21, Y26, Y53, Y69 or Y72) are derived from Y100.

X/Y100
X/Y21
X/Y26
X/Y53
X/Y69
X/Y72

Figure 1H.

Males were obtained from the following crosses:
C(1)DX, y^1, f^1 females x C(1;Y)1, y^1, Bar^1,Y* males (Y* being: Y100, Y21, Y26, Y53, Y69 or Y72)
C(1)DX/0 females were obtained by crossing C(1)DX, y^1, f^1 females directly with C(1;Y)1, y^1, Bar^1 males.

C(1)DX, y[1] f[1] (BL#64)/Y100
C(1)DX, y[1] f[1] (BL#64)/0
C(1)DX, y[1] f[1] (BL#64)/Y21
C(1)DX, y[1] f[1] (BL#64)/Y26
C(1)DX, y[1] f[1] (BL#64)/Y53
C(1)DX, y[1] f[1] (BL#64)/Y69
C(1)DX, y[1] f[1] (BL#64)/Y72

Figure 1I.

Males were obtained from the following crosses:
Canton-S females x C(1;Y)1, y^1, Bar^1,Y* males (Y* being: Y100, Y21, Y26, Y53, Y69 or Y72)
Note that C(1;Y)1, y^1, Bar^1 and each Y* chromosome were backcrossed eight generations into the laboratory's own Canton-S genetic stock. Y100 correspond to the Y chromosome of the following stock: BL#78567 and carries a Y-linked RFP transgene. All the Y deletions of our library (Y21, Y26, Y53, Y69 or Y72) are derived from Y100.

X/Y100
X/Y21
X/Y26
X/Y53
X/Y69
X/Y72

Figure 2.

Figure 2A.

Males were obtained from the following crosses:
C(1)M4,y^2 females x C(1;Y)1, y^1, Bar^1,Y* males (Y* being: Y100, Y21, Y26, Y53, Y69 or Y72)
C(1)M4,y^2/0 females were obtained by crossing C(1)M4,y^2 females directly with C(1;Y)1, y^1, Bar^1 males.

C(1)M4,y[2] (BL#1999)/0
C(1)M4,y[2] (BL#1999)/Y21

C(1)M4,y[2] (BL#1999)/Y26
C(1)M4,y[2] (BL#1999)/Y53
C(1)M4,y[2] (BL#1999)/Y69
C(1)M4,y[2] (BL#1999)/Y72
C(1)M4,y[2] (BL#1999)/Y100

Figure 2B.

Males were obtained from the following crosses:

y^1,w^{67c23},P{w[+mW.hs]=hsp26-pt-T}118E-25 females x C(1;Y)1, y^1, Bar^1,Y* males (Y* being: Y100, Y21, Y26, Y53, Y69 or Y72)

y^1,w^{67c23},P{w[+mW.hs]=hsp26-pt-T}118E-25/0 males were obtained by crossing y^1,w^{67c23},P{w[+mW.hs]=hsp26-pt-T}118E-25 females directly with C(1;Y)1, y^1, Bar^1 males.

y[1] w[67c23] P{w[+mW.hs]=hsp26-pt-T}118E-25 (BL#84091)/0
y[1] w[67c23] P{w[+mW.hs]=hsp26-pt-T}118E-25 (BL#84091)/Y21
y[1] w[67c23] P{w[+mW.hs]=hsp26-pt-T}118E-25 (BL#84091)/Y26
y[1] w[67c23] P{w[+mW.hs]=hsp26-pt-T}118E-25 (BL#84091)/Y53
y[1] w[67c23] P{w[+mW.hs]=hsp26-pt-T}118E-25 (BL#84091)/Y69
y[1] w[67c23] P{w[+mW.hs]=hsp26-pt-T}118E-25 (BL#84091)/Y72
y[1] w[67c23] P{w[+mW.hs]=hsp26-pt-T}118E-25 (BL#84091)/Y100

Figure 2C.

Males were obtained from the following crosses:

y^1,w^{67c23}; P{w[+mW.hs]=hsp26-pt-T}118E-10 females x C(1;Y)1, y^1, Bar^1,Y* males (Y* being: Y100, Y21, Y26, Y53, Y69 or Y72)

y^1,w^{67c23}; P{w[+mW.hs]=hsp26-pt-T}118E-10/0 males were obtained by crossing y^1,w^{67c23}; P{w[+mW.hs]=hsp26-pt-T}118E-10 females directly with C(1;Y)1, y^1, Bar^1 males.

y[1] w[67c23]/0;P{w[+mW.hs]=hsp26-pt-T}118E-10 (BL#84108)/+
y[1] w[67c23]/Y21;P{w[+mW.hs]=hsp26-pt-T}118E-10 (BL#84108)/+
y[1] w[67c23]/Y26;P{w[+mW.hs]=hsp26-pt-T}118E-10 (BL#84108)/+
y[1] w[67c23]/Y53;P{w[+mW.hs]=hsp26-pt-T}118E-10 (BL#84108)/+
y[1] w[67c23]/Y69;P{w[+mW.hs]=hsp26-pt-T}118E-10 (BL#84108)/+
y[1] w[67c23]/Y72;P{w[+mW.hs]=hsp26-pt-T}118E-10 (BL#84108)/+
y[1] w[67c23]/Y100;P{w[+mW.hs]=hsp26-pt-T}118E-10 (BL#84108)/+

Figure 2D.

In(1)w[m4]/Y100;+/+
In(1)w[m4]/0;+/+
In(1)w[m4]/Y100;;Su(var)3–9 (P[(ry+) Su(var)3–9 11 kb])/+
In(1)w[m4]/0;;Su(var)3–9 (P[(ry+) Su(var)3–9 11 kb])/+
In(1)w[m4]/Y100;Su(var)3-9[2] (BL#6210)/+
In(1)w[m4]/0;Su(var)3-9[2] (BL#6210)/+

Figure 2E.

Males were obtained from the following crosses:

In(1)w^{m4};Su(var)205^{5}/CyO females x C(1;Y)1, y^{1}, Bar^{1},Y* males (Y* being: Y100, Y21, Y53, or Y69)

In(1)w[m4]/Y100;+/+
In(1)w[m4]/0;+/+
In(1)w[m4]/Y100;Su(var)205[5] (BL#6234)/+
In(1)w[m4]/Y21;Su(var)205[5] (BL#6234)/+
In(1)w[m4]/Y53;Su(var)205[5] (BL#6234)/+
In(1)w[m4]/Y69;Su(var)205[5] (BL#6234)/+

Figure 3.

Figure 3A.

Males were obtained from the following crosses:
w^{1118}; esg-Gal4^{NP7397},UAS-GFP,Tub-Gal80^{TS}/CyO females x C(1;Y)1, y^{1}, Bar^{1},Y* males (Y* being: Y100, Y21, Y53, or Y69)
X/0 males were obtained by crossing w^{1118}; esg-Gal4^{NP7397},UAS-GFP,Tub-Gal80^{TS}/CyO females directly with C(1;Y)1, y^{1}, Bar^{1} males.

w[1118]/w[1118]; esg-Gal4[NP7397],UAS-GFP,Tub-Gal80[TS]/+
w[1118]/Y100; esg-Gal4[NP7397],UAS-GFP,Tub-Gal80[TS]/+
w[1118]/0; esg-Gal4[NP7397],UAS-GFP,Tub-Gal80[TS]/+
w[1118]/Y21; esg-Gal4[NP7397],UAS-GFP,Tub-Gal80[TS]/+
w[1118]/Y53; esg-Gal4[NP7397],UAS-GFP,Tub-Gal80[TS]/+
w[1118]/Y69; esg-Gal4[NP7397],UAS-GFP,Tub-Gal80[TS]/+

Figure 3B.

Males were obtained from the following crosses:
w^{1118}; esg-Gal4^{NP7397},UAS-GFP,Tub-Gal80^{TS}/CyO;UAS-Notch RNAi/TM6B females x C(1;Y)1, y^{1}, Bar^{1},Y* males (Y* being: Y100, Y21, Y53, or Y69)
X/0 males were obtained by crossing w^{1118}; esg-Gal4^{NP7397},UAS-GFP,Tub-Gal80^{TS}/CyO;UAS-Notch RNAi/TM6B females directly with C(1;Y)1, y^{1}, Bar^{1} males.

w[1118]/w[1118]; esg-Gal4[NP7397],UAS-GFP,Tub-Gal80[TS]/+;UAS-Notch RNAi (VDRC#GD27229)/+
w[1118]/Y100; esg-Gal4[NP7397],UAS-GFP,Tub-Gal80[TS]/+;UAS-Notch RNAi (VDRC#GD27229)/+
w[1118]/0; esg-Gal4[NP7397],UAS-GFP,Tub-Gal80[TS]/+;UAS-Notch RNAi (VDRC#GD27229)/+
w[1118]/Y21; esg-Gal4[NP7397],UAS-GFP,Tub-Gal80[TS]/+;UAS-Notch RNAi (VDRC#GD27229)/+
w[1118]/Y53; esg-Gal4[NP7397],UAS-GFP,Tub-Gal80[TS]/+;UAS-Notch RNAi (VDRC#GD27229)/+
w[1118]/Y69; esg-Gal4[NP7397],UAS-GFP,Tub-Gal80[TS]/+;UAS-Notch RNAi (VDRC#GD27229)/+
w[1118]/C(1,Y)1 (BL#4248); esg-Gal4[NP7397],UAS-GFP,Tub-Gal80[TS]/+;UAS-Notch RNAi (VDRC#GD27229)/+

Figure 3C/D/E.

Males were obtained from the following crosses:

Canton-S females x C(1;Y)1, y^1, Bar^1,Y* males (Y* being: Y100, Y21, Y53, or Y69)

Note that C(1;Y)1, y^1, Bar^1 and each Y* chromosome were backcrossed eight generations into the laboratory's own Canton-S genetic stock. Y100 correspond to the Y chromosome of the following stock: BL#78567 and carries a Y-linked RFP transgene. All the Y deletions of our library (Y21, Y26, Y53, Y69 or Y72) are derived from Y100.

X/0 males were obtained by crossing Canton-S females directly with C(1;Y)1, y^1, Bar^1 males.

XX
X/Y100
X/0
X/Y21
X/Y53
X/Y69

Figure 4.

Figure 4A.

Males were obtained from the following crosses:

Canton-S females x C(1;Y)1, y^1, Bar^1,Y* males (Y* being: Y100, Y21, Y53, or Y69)

Note that C(1;Y)1, y^1, Bar^1 and each Y* chromosome were backcrossed eight generations into the laboratory's own Canton-S genetic stock. Y100 correspond to the Y chromosome of the following stock: BL#78567 and carries a Y-linked RFP transgene. All the Y deletions of our library (Y21, Y26, Y53, Y69 or Y72) are derived from Y100.

X/0 males were obtained by crossing Canton-S females directly with C(1;Y)1, y^1, Bar^1 males.

X/Y100
X/0
X/Y21
X/Y53
X/Y69

Figure 4B.

X/Y100
X/0
C(1,Y)1 (BL#4248)/Y100

Figure 4C.

Males were obtained from the following crosses:

In(1)w^{m4};Su(var)205^5/CyO females x C(1;Y)1, y^1, Bar^1,Y* males (Y* being: Y100, Y53, or Y69)

X/0 males were obtained by crossing In(1)w^{m4};Su(var)205^5/CyO females directly with C(1;Y)1, y^1, Bar^1 males.

In(1)w[m4]/Y100;Su(var)205[5] (BL#6234)/+
In(1)w[m4]/0;Su(var)205[5] (BL#6234)/+
In(1)w[m4]/Y53;Su(var)205[5] (BL#6234)/+
In(1)w[m4]/Y69;Su(var)205[5] (BL#6234)/+

Figure 4D.

XX;TI{w[+mC]=mCherry}tra[KO.Cherry] (BL#67412)/+
X/Y;TI{w[+mC]=mCherry}tra[KO.Cherry] (BL#67412)/+
XX;TI{w[+mC]=mCherry}tra[KO.Cherry] (BL#67412)/TI{w[+mC]=mCherry}tra[KO.Cherry] (BL#67412)
X/Y;TI{w[+mC]=mCherry}tra[KO.Cherry] (BL#67412)/TI{w[+mC]=mCherry}traF

Extended Data Figure 1.

Extended Data Figure 1A.

w*;nubbin-Gal4 (BL#67086)/+;UAS-Cas9.P2 (BL#67086)/+
w*;nubbin-Gal4 (BL#67086)/+;UAS-Cas9.P2 (BL#67086)/UAS-gRNA x14
w*;nubbin-Gal4 (BL#67086)/+;UAS-Cas9.P2 (BL#67086)/UAS-gRNA x27
w*;nubbin-Gal4 (BL#67086)/+;UAS-Cas9.P2 (BL#67086)/UAS-gRNA x66

Extended Data Figure 1B.

w*/Dp(1;Y)B[S]Yy[+] (BL#3707);UAS-Cas9.P2 (BL#58985)/+;bam-Gal4/UAS-gRNA x14
w*/Dp(1;Y)B[S]Yy[+] (BL#3707);UAS-Cas9.P2 (BL#58985)/+;bam-Gal4/UAS-gRNA x27

Extended Data Figure 1C.

w*/TI{Disc\RFP[tdTom.3xP3]=attP}AByG (BL#78567);UAS-Cas9.P2 (BL#58985)/+;bam-Gal4/+
w*/TI{Disc\RFP[tdTom.3xP3]=attP}AByG (BL#78567);UAS-Cas9.P2 (BL#58985)/+;bam-Gal4/UAS-gRNA x14
w*/TI{Disc\RFP[tdTom.3xP3]=attP}AByG (BL#78567);UAS-Cas9.P2 (BL#58985)/+;bam-Gal4/UAS-gRNA x66
w*/TI{Disc\RFP[tdTom.3xP3]=attP}AByG (BL#78567);UAS-Cas9.P2 (BL#58985)/+;bam-Gal4/UAS-gRNA x223

Extended Data Figure 1D.

w*/TI{Disc\RFP[tdTom.3xP3]=attP}AByG (BL#78567);UAS-Cas9.P2 (BL#58985)/+;bam-Gal4/UAS-gRNA x14
w*/TI{Disc\RFP[tdTom.3xP3]=attP}AByG (BL#78567);UAS-Cas9.P2 (BL#58985)/+;bam-Gal4/UAS-gRNA x66
w*/TI{Disc\RFP[tdTom.3xP3]=attP}AByG (BL#78567);UAS-Cas9.P2 (BL#58985)/+;bam-Gal4/UAS-gRNA x223

Extended Data Figure 1E.

w*/TI{Disc\RFP[tdTom.3xP3]=attP}AByG (BL#78567);UAS-Cas9.P2 (BL#58985)/+;bam-Gal4/UAS-gRNA x223

Extended Data Figure 1F.

w/TI{Disc\RFP[tdTom.3xP3]=attP}AByG (BL#78567);UAS-Cas9.P2 (BL#58985)/+;bam-Gal4/UAS-gRNA x14*

w/TI{Disc\RFP[tdTom.3xP3]=attP}AByG (BL#78567);UAS-Cas9.P2 (BL#58985)/+;bam-Gal4/UAS-gRNA x66*

w/TI{Disc\RFP[tdTom.3xP3]=attP}AByG (BL#78567);UAS-Cas9.P2 (BL#58985)/+;bam-Gal4/UAS-gRNA x223*

Extended Data Figure 1G.

Males were obtained from the following crosses:

Canton-S females x C(1;Y)1, y^1, Bar^1,Y* males (Y* being: Y100, Y21, Y26, Y53, Y69 or Y72)

Note that C(1;Y)1, y^1, Bar^1 and each Y* chromosome were backcrossed eight generations into the laboratory's own Canton-S genetic stock. Y100 correspond to the Y chromosome of the following stock: BL#78567 and carries a Y-linked RFP transgene. All the Y deletions of our library (Y21, Y26, Y53, Y69 or Y72) are derived from Y100.

X/0 males were obtained by crossing Canton-S females directly with C(1;Y)1, y^1, Bar^1 males.

X/Y100

X/0

X/Y21

X/Y26

X/Y53

X/Y69

X/Y72

Extended Data Figure 1H.

Males were obtained from the following crosses:

Canton-S females x C(1;Y)1, y^1, Bar^1,Y* males (Y* being: Y100, Y26, or Y69)

Note that C(1;Y)1, y^1, Bar^1 and each Y* chromosome were backcrossed eight generations into the laboratory's own Canton-S genetic stock. Y100 correspond to the Y chromosome of the following stock: BL#78567 and carries a Y-linked RFP transgene. All the Y deletions of our library (Y21, Y26, Y53, Y69 or Y72) are derived from Y100.

X/0 males were obtained by crossing Canton-S females directly with C(1;Y)1, y^1, Bar^1 males

X/Y100

X/0

C(1;Y)1, y[1], Bar[1]/Y100

C(1;Y)1, y[1], Bar[1]/Y26

C(1;Y)1, y[1], Bar[1]/Y69

C(1;Y)1, y[1], Bar[1]/Y72

Extended Data Figure 1J.

w/TI{Disc\RFP[tdTom.3xP3]=attP}AByG (BL#78567);UAS-Cas9.P2 (BL#58985)/+;bam-Gal4/UAS-gRNA x14*

w/TI{Disc\RFP[tdTom.3xP3]=attP}AByG (BL#78567);UAS-Cas9.P2 (BL#58985)/+;bam-Gal4/UAS-gRNA x66*

w/TI{Disc\RFP[tdTom.3xP3]=attP}AByG (BL#78567);UAS-Cas9.P2 (BL#58985)/+;bam-Gal4/UAS-gRNA x223*

Extended Data Figure 1I.

Males were obtained from the following crosses:

Canton-S females x C(1;Y)1, y^{1}, Bar^{1},Y* males (Y* being: Y21, Y26, or Y69)

Note that C(1;Y)1, y^{1}, Bar^{1} and each Y* chromosome were backcrossed eight generations into the laboratory's own Canton-S genetic stock. Y100 correspond to the Y chromosome of the following stock: BL#78567 and carries a Y-linked RFP transgene. All the Y deletions of our library (Y21, Y26, Y53, Y69 or Y72) are derived from Y100.

X/0 males were obtained by crossing Canton-S females directly with C(1;Y)1, y^{1}, Bar^{1} males

X/0
X/Y21
X/Y26
X/Y69

Extended Data Figure 2.

y[1] w[*]/0;In(3L)BL1, P{w[enh]=HS-lacZ.scs}65E (BL#57370)/+
y[1] w[*]/Y53;In(3L)BL1, P{w[enh]=HS-lacZ.scs}65E (BL#57370)/+
y[1] w[*]/Y69;In(3L)BL1, P{w[enh]=HS-lacZ.scs}65E (BL#57370)/+
y[1] w[*]/Y72;In(3L)BL1, P{w[enh]=HS-lacZ.scs}65E (BL#57370)/+
y[1] w[*]/Y100;In(3L)BL1, P{w[enh]=HS-lacZ.scs}65E (BL#57370)/+

Extended Data Figure 3.

Extended Data Figure 3A/B/C/E.

XX
X/Y100
X/0

Extended Data Figure 3D.

y[1] w[*]/0;In(3L)BL1, P{w[enh]=HS-lacZ.scs}65E (BL#57370)/+
y[1] w[*]/Y53;In(3L)BL1, P{w[enh]=HS-lacZ.scs}65E (BL#57370)/+
y[1] w[*]/Y69;In(3L)BL1, P{w[enh]=HS-lacZ.scs}65E (BL#57370)/+
y[1] w[*]/Y100;In(3L)BL1, P{w[enh]=HS-lacZ.scs}65E (BL#57370)/+

Extended Data Figure 2F.

XX;TI{w[+mC]=mCherry}tra[KO.Cherry] (BL#67412)/+
XX;TI{w[+mC]=mCherry}tra[KO.Cherry] (BL#67412)/TI{w[+mC]=mCherry}tra[KO.Cherry] (BL#67412)
X/Y;TI{w[+mC]=mCherry}tra[KO.Cherry] (BL#67412)/+
X/Y;TI{w[+mC]=mCherry}tra[KO.Cherry] (BL#67412)/TI{w[+mC]=mCherry}traF
