# Supplementary material for: Y chromosome toxicity does not contribute to sex-specific differences in longevity
Source: Nat Ecol Evol. 2023 Jun 12;7(8):1245–56. doi: 10.1038/s41559-023-02089-7 (PMC10406604; doi:10.1038/s41559-023-02089-7)
Supplement: Supplementary file 2 — Reporting Summary [file 41559_2023_2089_MOESM2_ESM.pdf]

nature portfolio

Corresponding author(s): Delanoue Renald
Hudry bruno

Last updated by author(s): Mar 17, 2023

Reporting Summary

Nature Portfolio wishes to improve the reproducibility of the work that we publish. This form provides structure for consistency and transparency in reporting. For further information on Nature Portfolio policies, see our Editorial Policies and the Editorial Policy Checklist.

Statistics

For all statistical analyses, confirm that the following items are present in the figure legend, table legend, main text, or Methods section.

n/a	Confirmed	
☐	☒	The exact sample size (*n*) for each experimental group/condition, given as a discrete number and unit of measurement
☐	☒	A statement on whether measurements were taken from distinct samples or whether the same sample was measured repeatedly
☐	☒	The statistical test(s) used AND whether they are one- or two-sided<br>*Only common tests should be described solely by name; describe more complex techniques in the Methods section.*
☒	☐	A description of all covariates tested
☐	☒	A description of any assumptions or corrections, such as tests of normality and adjustment for multiple comparisons
☒	☐	A full description of the statistical parameters including central tendency (e.g. means) or other basic estimates (e.g. regression coefficient) AND variation (e.g. standard deviation) or associated estimates of uncertainty (e.g. confidence intervals)
☐	☒	For null hypothesis testing, the test statistic (e.g. *F*, *t*, *r*) with confidence intervals, effect sizes, degrees of freedom and *P* value noted<br>*Give P values as exact values whenever suitable.*
☒	☐	For Bayesian analysis, information on the choice of priors and Markov chain Monte Carlo settings
☒	☐	For hierarchical and complex designs, identification of the appropriate level for tests and full reporting of outcomes
☒	☐	Estimates of effect sizes (e.g. Cohen's *d*, Pearson's *r*), indicating how they were calculated

Our web collection on statistics for biologists contains articles on many of the points above.

Software and code

Policy information about availability of computer code

Data collection	*Provide a description of all commercial, open source and custom code used to collect the data in this study, specifying the version used OR state that no software was used.*
Data analysis	All statistical analyses were carried out using ImageJ (2015), Microsoft Excel (version 16.16.27), and GraphPad Prism 9.2.0 (283).

For manuscripts utilizing custom algorithms or software that are central to the research but not yet described in published literature, software must be made available to editors and reviewers. We strongly encourage code deposition in a community repository (e.g. GitHub). See the Nature Portfolio guidelines for submitting code & software for further information.

Data

Policy information about availability of data

All manuscripts must include a data availability statement. This statement should provide the following information, where applicable:

- Accession codes, unique identifiers, or web links for publicly available datasets
- A description of any restrictions on data availability
- For clinical datasets or third party data, please ensure that the statement adheres to our policy

All data is available in the main text or the supplementary data. Materials generated for the study are available from the corresponding authors on request.

March 2021

Human research participants

Policy information about studies involving human research participants and Sex and Gender in Research.

Reporting on sex and gender	n/a
Population characteristics	*Describe the covariate-relevant population characteristics of the human research participants (e.g. age, genotypic information, past and current diagnosis and treatment categories). If you filled out the behavioural & social sciences study design questions and have nothing to add here, write "See above."*
Recruitment	*Describe how participants were recruited. Outline any potential self-selection bias or other biases that may be present and how these are likely to impact results.*
Ethics oversight	*Identify the organization(s) that approved the study protocol.*

Note that full information on the approval of the study protocol must also be provided in the manuscript.

Field-specific reporting

Please select the one below that is the best fit for your research. If you are not sure, read the appropriate sections before making your selection.

☒ Life sciences ☐ Behavioural & social sciences ☐ Ecological, evolutionary & environmental sciences

For a reference copy of the document with all sections, see nature.com/documents/nr-reporting-summary-flat.pdf

Life sciences study design

All studies must disclose on these points even when the disclosure is negative.

Sample size	No a priori sample-size calculation was performed, sample size was set according to the reproducibility of each experiment. ARRIVE guidelines had been followed for the study and the maximum number of replicates were used for each experiment above which additional replicates did not alter the statistical significance.
Data exclusions	No data were excluded from the analyses.
Replication	Each unique experiments were repeated at least three independent times (n). "n" refers to the number of biological replicates for each experimental groups. The number of technical replicates, the experimental units, and number of experimental units allocated to each group are indicated for all experiments in the Figures and/or in the Figure legends. All attempts at replication were successful.
Randomization	Samples were randomly selected for analysis and were randomly allocated into experimental groups.
Blinding	All experiments were conducted single-blind. Each experimental group were given numbers prior to dissection and analysis. Only after data were recorded experimental numbers were brought with the genotypes/treatment groups.

Reporting for specific materials, systems and methods

We require information from authors about some types of materials, experimental systems and methods used in many studies. Here, indicate whether each material, system or method listed is relevant to your study. If you are not sure if a list item applies to your research, read the appropriate section before selecting a response.

Materials & experimental systems

n/a	Involved in the study	
☐	☒	Antibodies
☒	☐	Eukaryotic cell lines
☒	☐	Palaeontology and archaeology
☐	☒	Animals and other organisms
☒	☐	Clinical data
☒	☐	Dual use research of concern

Methods

n/a	Involved in the study	
☒	☐	ChIP-seq
☒	☐	Flow cytometry
☒	☐	MRI-based neuroimaging

Antibodies

Antibodies used	The following antibodies were used: mouse anti-Phospho-gammaHis2Av (1/250) (DSHB), rabbit anti-Phospho-histone H3 (Ser10) (1/500) (9701 Cell Signaling), chicken anti-GFP (1/10000) (ab13970 Abcam), mouse anti-Histone H3 (di-methyl K9) (1/500) (ab1220 Abcam), rabbit anti-Histone H3 (tri-methyl K9) (1/1000) (ab8898 Abcam), rabbit anti-Stellate (1/1000) (gift from W.E. Theurkauf26).
Validation	mouse anti-Phospho-gammaHis2Av (1/250) (DSHB): validation in The development of a monoclonal antibody recognizing the Drosophila melanogaster phosphorylated histone H2A variant (γ-H2AV). Hawley RS. G3 (Bethesda, Md.) 3.9 (2013 Sep 4): 1539-43.<br>Rabbit anti-phospho-histone H3 Ser10 (9701L, Cell Signalling Technology, validation: Cell Signalling Technology for W, IHC-P, IF-IC, Reacts with-D. melanogaster)<br>chicken anti-GFP (1/10000) (ab13970 Abcam, validation: Abcam suitable for ELISA, IHC-Fr, ICC, IHC-P, IP, WB, IHC-FoFr, IHC-FrFl, Electron Microscopy, Reacts with: Species independent)<br>mouse anti-Histone H3 (di-methyl K9) (1/500) (ab1220 Abcam, validation: Abcam suitable for: ICC/IF, WB, ELISA, IHC-P, ChIP, reacts with: Cow, Human, Arabidopsis thaliana, Drosophila melanogaster, Rice, Recombinant fragment)<br>rabbit anti-Histone H3 (tri-methyl K9) (1/1000) (ab8898 Abcam, validation: Abcam suitable for: WB, IHC-P, ICC, ChIP, reacts with: Mouse, Cow, Human)<br>rabbit anti-Stellate (1/1000) (gift from W.E. Theurkauf, validation in Klattenhoff, C. et al. Drosophila rasiRNA Pathway Mutations Disrupt Embryonic Axis Specification through Activation of an ATR/Chk2 DNA Damage Response. Dev. Cell 12, 45–55 (2007))

Animals and other research organisms

Policy information about studies involving animals; ARRIVE guidelines recommended for reporting animal research, and Sex and Gender in Research

Laboratory animals	UAS transgenes: UAS-Cas9 (BDSC: 67086 and BDSC: 58985), UAS-gRNA x14 (this study), UAS-gRNA x27 (this study), UAS-gRNA x66 (this study), UAS-gRNA x223 (this study), UAS-NotchRNAi (VDRC#GD 27229), and Su(var)3–9 11 kb (generated in32, gift from G. Reuter).<br>Mutants: Su(var)3-92 (BDSC: 6210), Su(var)2055 (BDSC: 6234), traKO (BDSC: 67412), and traF (generated in47).<br>Gal4 drivers: nubbin-Gal4 (BDSC: 67086), eyeless-Gal4 (gift from P. Meier), bam-Gal4 (gift from M. Amoyel), and esg-Gal4NP7397, UAS-GFP, Tub-Gal80TS chromosome (gift from J. de Navascués).<br>Reporters: w118E-25 (BDSC: 84091), w118E-10 (BDSC: 84108), and LacZIn(3L)BL1 (BDSC: 57370).<br>Sex chromosomes: YBar (BDSC: 81622), YRFP (BDSC: 78567), YBar+yellow (BDSC: 3707), Y21 (this study), Y26 (this study), Y53 (this study), Y69 (this study), Y72 (this study), C(1)DX (BDSC: 64), C(1)M4 (BDSC: 1999), C(1)RM (BDSC: 4248) and C(1,Y)1 (BDSC: 4248).
Wild animals	No wild animals were used in the study.
Reporting on sex	Both sexes were included. Sex-based analyses were performed.
Field-collected samples	No field collected samples were used in the study.
Ethics oversight	*Identify the organization(s) that approved or provided guidance on the study protocol, OR state that no ethical approval or guidance was required and explain why not.*

Note that full information on the approval of the study protocol must also be provided in the manuscript.
